# Skin Disorders among Elder Patients in a Referral Center in Northern Iran (2011)

**DOI:** 10.1155/2013/193205

**Published:** 2013-07-08

**Authors:** Abbas Darjani, Zahra Mohtasham-Amiri, Kiarash Mohammad Amini, Javad Golchai, Shahryar Sadre-Eshkevari, Narges Alizade

**Affiliations:** ^1^Department of Dermatology, Medical Faculty of Guilan University, Rasht, Iran; ^2^Department of Preventive and Community Medicine, Medical Faculty of Guilan University, 4163545851 Rasht, Iran

## Abstract

*Background*. Geriatric health care has become a worldwide concern, but a few statistical studies were carried out about skin diseases in this age group. In this study, we set out to determine the frequency as well as the age and gender distribution of dermatological diseases in geriatric patients. *Materials and Methods*. In a cross-sectional study, all patients over 60 years who were accepted to department of dermatology in north of Iran participated in this study. Baseline information and clinical examination were done by a group of dermatologists. Biopsy, Pathological and laboratory methods were used in order to confirm the diagnosis. *Results*. 440 patients were accepted to the department that 232 patients were male (52.7%). Benign neoplasm was as the common skin disease among patients (65%), followed by erythemo-squamous (35.3%) and precancerous lesions (26.1%). The most common precancerous lesion was actinic keratosis (24.3%). BCC by 8.8% was the most prevalent skin carcinoma. Pruritus was the common problem in other dermatological disease (22%). *Conclusion*. Skin disorders especially precancerous lesion are among those important health problems in elderly patients in this geographic area. Increasing general awareness about risk factors of these diseases and doing more researches in other regions are highly recommended.

## 1. Introduction

From 2000 until 2050, the world's population aged 60 and over will more than triple from 600 million to 2 billion. Most of this increase will occur in less developed countries, where the number of older people will rise from 400 million in 2000 to 1.7 billion by 2050 [1].

 In the most countries of the world, the proportion of people of over 60 years old is growing faster than any other age group: this fact is happening as a result of both longer life expectancy and also of what we can determine as the declining fertility rates. This population ageing can be seen as a success story for public health policies and for socioeconomic development; on the other hand, it also challenges society to adapt, in order to maximize the health and functional capacity of older people [2].

The United Nations statistical projections demonstrate a rapid growth of elderly population in Iran. While the proportion of people with 60 years old age and above in Iran was 5.4 percent in 1975, it will increase to 10.5 percent in 2025 and 21.7 percent in 2050 [3]. According to the Census of Population and Housing 2006 of Iran, population of the Guilan province in north of Iran was 2404861 people with 242850 (10.09%) aged 60 years and above. Indeed, the province of Guilan has the highest elderly rate in Iran [4].

In aging, a decline in the regular functions of skin is observed, including cell replacement capacity, barrier function, chemical clearance capacity, sensory perception, mechanical protection, wound healing, immune responsiveness, thermoregulation, sweat production, sebum production, vitamin D production, and capacity to repair DNA. As a result, some inevitable changes, such as roughness, wrinkling, and laxity of the skin, and atypical presentations of dermatologic diseases are observed in elderly patients [5].

Geriatric health care has absorbed a worldwide attention, but few statistical studies were carried out about skin diseases in this age group. 30 years ago, the American HANES survey demonstrated that the frequency of skin disorders increases with age so that at the age of 70 some 70% had a significant skin condition and many others had multiple skin problems [6]. 

In this study, we attempted to determine the frequency as well as the age and gender distributions of dermatological diseases in geriatric patients who attended dermatologic center of an educational hospital in Rasht, Iran.

## 2. Methods and Materials

In a cross-sectional study, 440 patients over 60 years old have participated in this study between March 2010 and March 2011. Informed consent was received from all patients. Baseline information on sociodemographic variables, past medical history, and medication was all gathered by medical staff during a face-to-face interview before doing any examination. Each patient was examined carefully by two dermatologists. Biopsy, pathological, and laboratory methods were used to confirm the diagnosis of suspected lesions or disease. The diseases were categorized into seven different groups including erythematosquamous diseases (such as psoriasis, lichen planus, seborrheic dermatitis, contact dermatitis, paederus dermatitis, stasis dermatitis, lichen simplex chronics, and pilaris rubra pityriasis), infectious diseases (fungal, bacterial, and viral infections, infestations), benign neoplasm (pillar cyst, keloids, lipoma, seborrheic keratosis, pyogenic granuloma, epidermal cyst, keratoacantom, and skin tag), precancerous lesions (leukoplakia, actinic keratosis, and bowen), skin cancer (basal cell carcinoma BCC, Squamish cell carcinoma SCC, mycosis fungoides, and kaposis sarcoma), age-related skin changes (xerosis, senile lentigo, senile comedon, angioma, and nail ridging), and the others (leg ulcer, insect bite, sarcoidosis, ingrowing toe nail, corn, vasculitis, and vitiligo). 

Data analysis was carried out with the SPSS Software, version 18. Descriptive statistics for the prevalence of skin disease were calculated, and gender differences in skin disease prevalence are investigated using a chi-square testing all the analyses; a *P*-value of <0.05 was considered statistically significant.

## 3. Results

During the study period, 4231 patients were accepted to the department. Among the patients who were enrolled in this study, 440 persons (10.4%) were older than 60 years; meanwhile 232 patients were males (52.7%) and the others were females (47.3%). Most of them were in the age group 60–69 years (57%), and 10% were above 80 years. Hypertension (27%), diabetes mellitus (18%), and heart disease (11%) were the most prevalent underlying diseases. Most of the participants were farmers (41%), retirees (13%), drivers (10%), and fishermen (4%), respectively.

Benign neoplasm was the common skin disease among patients (65%), followed by erythematosquamous (35.3%), and precancerous lesions (26.1%). Most of the patients (76%) had more than one skin disease. Age-related skin changes were seen in all patients (Table 1).

The most frequent diseases of erythematosquamous diseases were defined as dermatitis (16.6%), psoriasis (12.3%), lichen planus (5.45%) and pilaris rubra pityriasis (1.1%). Fungal infections (tinea, candidiasis) were the most common infectious diseases (8.2%) followed by viral infections (herpes zoster) (4.5%) and infestations (scabies) (4.3%). The most common precancerous lesion was actinic keratosis (24.3%). BCC by 8.8% was the most prevalent skin carcinoma. Skin tag (48.8%) and seborrheic keratosis (8%) were the most common benign neoplasm. 69% of patients with skin tag were females. Pruritus was the common problem in other dermatological diseases (22%). The most frequent diseases according to age groups were shown in Table 2.

## 4. Discussion

Dermatologic clinic of Razi hospital is the referral clinic of dermatology in Guilan that covers in-hospital and outpatients dermatologic diseases in the most part of northern area of Iran. Since the majority of outpatient skin disorders are referred to private section and are not reported to the public official sector, the hospital-base data is the only accessible source. On the other hand, it is possible that differences between the distributions of the diseases were seen here with other community-based studies.

The majority of patients were exposed to sunlight due to their jobs. Also more than 62% of the patients had at least one underlying disease that was under medication to treat. Some of these medications can cause or aggravate skin diseases.

In our study, the above mentioned benign neoplasm group was the most common skin disease defined among the patients who have been taken into consideration; this disease affected almost two-thirds of them (65%). Seborrheic keratosis (8%) was the most common benign neoplasm. This rate is different from other studies of elderly populations, where its prevalence of 1.7% to 85% has been recorded [5, 7–11]. Seborrheic keratosis is the most common benign epithelial tumor of adulthood. These differences may be related to different settings of the participants (population-base, clinic-base or nursing home), different climate, and different jobs of participants. It seems that sunlight exposure may play a role because seborrheic keratoses are common on sun exposed areas such as the back, arms, face, and neck [12, 13]. Two-thirds of patients with skin tag were females. Obesity and overweight (even temporary increases in weight) dramatically increase the chances of having skin tags [14]. Unfortunately, we did not record weights and heights of patients to reveal the relation between skin tag and obesity in our patients.

In our study, actinic keratosis was the most common skin disease after age-related skin changes with 24.3% prevalence rate. Actinic keratosis is a precursor lesion to squamous cell carcinoma, and lifetime sun exposure is an important risk factor for it, and various studies cite a <1–20% risk of transformation to squamous cell carcinoma (SCC) for an individual lesion over the course of a year [15]. This rate is higher than the reported rates of this disease in Tehran [16] and Italy [17] but consistent with other studies in old aging in Taiwan, Croatia, and Australia with, respectively, 22.4% (8), 22.3% (10) and 25% [11]. This similar rate may be due to similar climate in these area, and exposure to sunlight. 

Pruritus with high rate as 22% was a common problem in our study. Pruritus in the elderly can be caused by a variety of dermatological and systemic conditions, but the most common cause is dry skin [18]. Low humidity combined with hot showers and overuse of soaps results in dryness and cracking of the skin. This rate is different from other studies of elderly populations, where its prevalence of 6.4% in Tunisia [9], 8.8%–11.5% in Turkey [5, 7], 14.2% in Taiwan [8], 18.9% in Italy [17], and 49.6% in India [19] has been recorded [5, 7–11].

Xerosis also was reported from 11.6% of patients. This disease was shown as 1.5% in Japan [20], 5.4% in Turkey [7], 18.2% in Hong Kong [19], 28% in Tehran [16], 29.5% in Australia [21], 58.3% in Taiwan [22], and 77% in a systematic review [11]. Differences in humidity and lifestyle can describe the wide difference of these complaints in the elderly.

Dermatitis was another common problem in this study that was affected 16.6%. Other studies have shown different rates from 1.5% to 58.7% [5, 7, 10, 19–24]. Contact dermatitis (most common form of dermatitis) is a delayed-type hypersensitivity reaction to an antigen (allergen) that contacts the skin; indeed, the higher incidence of dermatitis in this age group may happen due to more contact with the environmental and physical factors.

Cutaneous malignant tumors were found in 15.4% of prominently, in male patients in comparison with female (*P* value <0.001). BCC was diagnosed in 8.8% that was the most prevalent skin carcinoma. Basal cell carcinoma is the most common skin cancer (~75%) and is related to chronic ultraviolet light exposure.

The proportion of skin tumor cases in this study was much higher than other studies which have been done in western and eastern parts of the country [8, 9, 19, 21–23]. Type of the clinic as referral clinic and jobs of patients may be the most causes of this high rate of malignancy. 

Fungal infections were the most common infectious diseases in our study that were found out in 8.2%. Other studies showed different rates from 4.4% to 61.6% [8, 9, 19–23]. Fungal infection depends on underlying diseases such as diabetes, bedridden status, and also hygiene level of patients. It seems that infection diseases are more prevalent among nursing home care patients in comparison with community-dowelling patients. 

Overall, our research supports the opinion that skin diseases are a common and inevitable consequence of aging. A lifetime of solar radiation exposure, combined with intrinsic changes in the dermal structures, predisposes geriatric individual to a wide variety of skin diseases; we witnessed many of them during this research.

## Figures and Tables

**Table 1:** The distributions of all skin diseases according to gender.

Disease	Total *n* (440)		Male *n* (232)		Female *n* (208)		*P* value
	*n*	%	*n*	%	*n*	%	
Age-related skin changes	440	100	232	100	208	100	NS
Erythematosquamous diseases	156	35.3	84	36.2	72	34.6	NS
Infectious diseases	89	20.2	40	17.2	49	23.6	NS
Benign neoplasm	286	65	149	64.2	137	65.7	NS
Precancerous lesions	115	26.1	62	26.7	53	25.5	NS
Skin carcinomas	68	15.4	47	20.3	21	10.1	0.001
Others	223	50.7	114	49.1	109	52.4	NS

**Table 2:** The most prevalent skin diseases according to age groups.

Disease	Age group					
	60–69 years no. (250)		70–79 years no. (144)		≥80 years no. (46)	
	No.	%	No.	%	No.	%
Age-related nails changes	209	83.6	125	86.8	40	86.9
Senile angioma	133	53.2	76	52.7	22	47.8
Lentigo	113	45.2	68	47.2	26	56.5
Skin tag	118	47.2	70	48.6	27	58.7
Actinic keratosis	61	24.4	36	25	10	21.7
Pruritus	52	20.8	33	22.9	12	26
Dermatitis	40	16	20	13.8	13	28.3
Psoriasis	36	14.4	13	9	5	10.9
Xerosis	30	12	14	9.7	7	15.2
BCC	20	8	13	9	6	13
Fungal infection	20	8	10	6.9	6	13
Pemphigus vulgaris	25	10	10	6.9	2	4.3
Seborrheic keratosis	19	7.6	12	8.3	5	10.9
Lichen planus	15	6	6	4.2	3	6.5
Herpes zoster	11	4.4	6	4.2	3	6.5
Scabies	10	4	5	3.5	4	8.7
Callosity	10	4	5	3.5	3	6.5
SCC	10	4	5	3.5	2	4.4
Leg ulcer	8	3.2	6	4.2	3	6.5
Mycosis fungoides	5	2	3	2.1	1	2.2
Keratoacantom	5	2	1	0.7	1	2.2
